# Added Sugar Intake Is Associated with Altered Plasma miR-143-5p and miR-223-3p Expression and Fasting Glucose Concentrations in Children from Mexico City

**DOI:** 10.3390/biom16081100

**Published:** 2026-07-28

**Authors:** Alejandro Castañeda-López, María F. Velázquez-Rodríguez, Leonor Jacobo-Albavera, María Fernanda Pérez-Hernández, José de Jesús Peralta-Romero, Sofía Barragán-Vázquez, Claudia I. Ramírez-Silva, Andrés Rocha-Aguado, Fengyang Huang, Fausto Sánchez-Muñoz, Miguel Cruz, Adrián Hernández-Díazcouder

**Affiliations:** 1 Unidad de Investigación Médica en Bioquímica, Hospital de Especialidades, Centro Médico Nacional Siglo XXI, Instituto Mexicano del Seguro Social (IMSS), Mexico City 06720, Mexico; alex.qfb9@gmail.com (A.C.-L.);; 2 Departamento de Ciencias de La Salud, Universidad Tecnológica de México (UNITEC) Campus Los Reyes, Los Reyes 56400, State of Mexico, Mexico; 3 Laboratorio de Genómica de Enfermedades Cardiovasculares, Instituto Nacional de Medicina Genómica (INMEGEN), Mexico City 14610, Mexico; 4 Programa de Doctorado en Ciencias Biomédicas, Universidad Nacional Autónoma de México (UNAM), Mexico City 04510, Mexico; 5 Nutritional Epidemiology Group, School of Food Science & Nutrition, University of Leeds, Woodhouse Ln, Woodhouse, Leeds LS2 9 JT, UK; 6 Instituto Nacional de Salud Pública, Cuernavaca 62100, Mexico; 7 OOAD DF Norte del Instituto Mexicano del Seguro Social, Unidad de Medicina Familiar No. 23, Mexico City 07070, Mexico; 8 Laboratorio de Investigación en Obesidad y Asma, Hospital Infantil de México Federico Gómez, Mexico City 06720, Mexico; 9 Departamento de Fisiología, Instituto Nacional de Cardiología Ignacio Chávez, Mexico City 14080, Mexico

**Keywords:** added sugar, circulating microRNAs, childhood obesity, cardiometabolic factors, insulin resistance, miR-143-5p, miR-223-3p

## Abstract

**Background:** Childhood obesity is a growing health concern and added sugar intake has been associated with increased obesity risk. Emerging evidence suggests that miR-143-5p and miR-223-3p are involved in metabolic regulation. Therefore, this study evaluated their plasma expression according to added sugar intake and their association with cardiometabolic risk parameters. **Methods:** This cross-sectional study included 91 children aged 6–12 years, comprising 44 with normal weight and 47 with overweight/obesity. Anthropometric measurements, clinical characteristics, and dietary added sugar intake were assessed. Plasma expression of miR-143-5p and miR-223-3p was quantified by RT-qPCR. **Results:** Compared with low added sugar intake, high added sugar intake was associated with lower plasma miR-143-5p expression and higher miR-223-3p expression. Both miR-143-5p and miR-223-3p expression levels were higher in children with overweight/obesity and insulin resistance than in their respective counterparts. In adjusted analyses, neither miRNA was associated with cardiometabolic risk parameters. However, added sugar intake was positively associated with miR-223-3p expression (β = 0.188, 95% CI: 0.022, 0.354) and glucose levels (β = 1.944, 95% CI: 0.133, 3.755). **Conclusions:** These findings suggest that higher added sugar intake is associated with altered circulating miRNA expression and higher fasting glucose concentrations in school-aged children.

## 1. Introduction

Childhood obesity represents a growing global health challenge, given that overweight and obesity during childhood frequently persist into adulthood [1]. Epidemiological evidence has shown a marked worldwide rise in the number of children and adolescents living with overweight and obesity. In particular, the prevalence of obesity among children and adolescents aged 5–19 years increased more than threefold globally, from 2.0% in 1990 to 8.7% in 2025, and is projected to reach 11.9% by 2040 [2,3]. In addition, in Mexico, the combined prevalence of overweight and obesity among children aged 5–11 years has been reported to be 36.6% [4]. Childhood obesity is associated with the early development of cardiometabolic risk factors, including hypertension, dyslipidemia, insulin resistance, and impaired glucose metabolism [5].

Among the dietary factors associated with this condition, higher added sugar intake has been linked to weight gain and an increased risk of obesity in children [6,7,8]. Added sugars are primarily consumed as sucrose and high-fructose corn syrup through sugar-sweetened beverages, including soft drinks, sweetened teas, and fruit drinks, which represent major dietary sources of added sugars among children and adolescents [9]. High consumption of these sugars has also been associated with dyslipidemia, insulin resistance, and elevated uric acid levels, all of which are associated with an increased risk of obesity, metabolic dysfunction-associated steatotic liver disease, and other cardiometabolic complications [10]. In Mexico, the 2020–2022 National Health and Nutrition Survey (ENSANUT) [11] reported that added sugar intake among children aged 5 to 11 years was approximately 50 g/day, representing 12% of total energy intake. In addition, 66.9% of school-aged children consumed more than 10% of their daily energy intake from added sugar [11]. In this context, the World Health Organization (WHO) recommends reducing free sugar intake to less than 10% of total energy intake.

Emerging studies suggest that epigenetic factors such as microRNAs (miRNAs) play a role in metabolic regulation. MiRNAs are small, endogenous, single-stranded non-coding RNAs that play an important role in the post-transcriptional regulation of gene expression through their interaction with the 3′ untranslated region (3′UTR) of target messenger RNAs (mRNAs) [12]. miRNAs are released from cells into the circulation [13], where extracellular circulating miRNAs contribute to the regulation of whole-body metabolism through intercellular communication [14]. Circulating miRNAs have gained attention as novel biomarkers of obesity and related metabolic disorders [14,15].

Accumulating evidence indicates thar chronic energy surplus and dietary factors can modulate the expression of circulating and tissue-specific miRNAs involved in metabolic homeostasis. Evidence from both animal models and human studies has shown that high-fat and high-sugar diets alter the expression of circulating and tissue-specific miRNAs associated with adipogenesis, lipid metabolism, insulin signaling, and inflammation, supporting their potential use as biomarkers of metabolic dysfunction [16,17,18]. Several studies have highlighted the important role of miR-143-5p in lipid metabolism, adipogenesis, and IR [19]. Experimental studies have shown that obesity-induced overexpression of miR-143 impairs insulin-stimulated AKT activation through the downregulation of oxysterol-binding protein-related protein 8, thereby contributing to impaired glucose metabolism and insulin resistance [20]. Also, this miRNA promotes lipid accumulation in adipocytes [21]. miR-223-3p is involved in the regulation of inflammatory signaling and glucose metabolism [22]. Experimental evidence suggests that miR-223-3p modulates inflammatory signaling through the regulation of the NLRP3 inflammasome, thereby contributing to obesity-related metabolic dysfunction and insulin resistance [23]. Moreover, experimental studies have reported that these miRNAs may be regulated by high-sucrose and high-fructose diets [24,25]. In this context, the analysis of plasma miRNA expression provides a practical approach for investigating molecular alterations associated with dietary factors and obesity in children. Thus, the present study aimed to evaluate the association between added sugar intake and plasma expression of miR-143-5p and miR-223-3p, as well as their relationship with cardiometabolic risk parameters.

## 2. Materials and Methods

### 2.1. Participants in the Study

The study population consisted of 91 unrelated children from Mexico City, aged 6 to 12 [44 normal weight (NW) and 47 with overweight and obesity (Ow/Ob)]. Participants were recruited at Family Medicine Unit No. 23 of the Instituto Mexicano del Seguro Social in Mexico City. The study was conducted to evaluate the association of added sugar intake with miRNA expression, obesity, and cardiometabolic risk factors. Exclusion criteria included acute infectious diseases, chronic medical conditions, participation in a weight loss intervention, and fasting plasma glucose concentrations exceeding 126 mg/dL.

### 2.2. Ethics Statement

The research protocol was reviewed and approved by the Ethics Committee of the Instituto Mexicano del Seguro Social (CONBIO-ETICA-09-CEI-009-20160601; approval No. R-2024-785-061) on 28 November 2025. All study procedures complied with the principles outlined in the Declaration of Helsinki. Written informed consent was provided by the parents or legal guardians of all participants, and written assent was obtained from the children before participation.

### 2.3. Anthropometric Assessment

According to standardized protocol, the following anthropometric measurements were performed to ensure accuracy, consistency, and data reliability. The anthropometric measurements were performed by trained personnel using calibrated instruments according to standardized procedures.

Body weight, height, waist circumference, and hip circumference were measured for all participants during a single study visit. Body weight was determined to the nearest 0.1 kg using a calibrated digital scale (Seca, Hamburg, Germany), with participants wearing light clothing and no shoes. Height was measured to the nearest 0.1 cm using a portable stadiometer (Seca 225, Hamburg, Germany), with participants standing barefoot in the Frankfurt horizontal plane. Waist circumference was measured to the nearest 0.1 cm using a non-elastic measuring tape positioned immediately above the iliac crest at the end of a normal expiration. Hip circumference was obtained using the same measuring tape at the widest portion of the buttocks. All anthropometric measurements were performed according to standardized procedures and recorded immediately after assessment. Body mass index (BMI) was calculated as weight (kg) divided by height squared (m^2^). BMI-for-age z-scores were calculated using WHO AnthroPlus software, and nutritional status was classified according to the World Health Organization (WHO) growth reference for children and adolescents [26]. Nutritional status was classified according to the WHO growth reference for children and adolescents aged 5–19 years based on BMI-for-age z-scores. Participants with BMI-for-age z-scores between −2 and +1 SD were classified as having normal weight, whereas those with z-scores > +1 SD were classified as having overweight/obesity [26].

### 2.4. Biochemical Assessment

Following an overnight fast of at least 8 h, venous blood was collected during the morning to determine fasting glucose, insulin, low-density lipoprotein cholesterol (LDL-C), high-density lipoprotein cholesterol (HDL-C), total cholesterol (Total CHO), and triglyceride (TG) concentrations. Fasting serum glucose, low-density lipoprotein cholesterol (LDL-C), high-density lipoprotein cholesterol (HDL-C), total cholesterol (Total CHO), and triglyceride (TG) concentrations were determined by enzymatic colorimetric assays using the ILab 350 Clinical Chemistry System (Instrumentation Laboratory IL, Barcelona, Spain). Serum insulin concentrations were quantified with the Elecsys Insulin Assay on a Cobas e411 immunoassay analyzer (Roche Diagnostics GmbH, Mannheim, Germany). Fasting glucose and insulin concentrations were used to calculate the homeostasis model assessment of IR (HOMA-IR) using the following formula: fasting insulin (µU/mL) × fasting glucose (mg/dL)/405. IR was defined as HOMA-IR ≥ 3.16, based on a cutoff previously proposed for children and adolescents with obesity [27,28].

### 2.5. Assessment of Added Sugar Intake

A semi-quantitative food frequency questionnaire (FFQ) was used to assess dietary added sugar intake over the past month [29]. The FFQ was designed based on the Mexican ENSANUT for school-aged children, developed by the National Institute of Public Health, as a reference. For food items newly incorporated into the FFQ because of their relevance to the study population, reference portion sizes were derived from 24 h dietary recall data collected in school-aged children participating in ENSANUT, and household measures were used to standardize portion size estimation. Added sugar intake was calculated using an algorithm based on the methodology described by Louie et al. [30], adapted by our research group, together with data from the Mexican Food Composition Database (BAM) [31]. The FFQ data were processed according to the same analytical procedures applied for estimating food and nutrient intake in the ENSANUT FFQ [32]. Children were classified as having high added sugar intake when consumption was ≥50 g/day and low added sugar intake when consumption was <50 g/day. This cutoff was defined based on a 2000 kcal diet, which corresponded approximately to the average total energy intake of the study population.

### 2.6. miRNA Expression in Plasma

RNA extraction from 150 µL of plasma was performed using the miRNeasy Serum/Plasma Kit (Qiagen, Waltham, Hilden, Germany), according to the manufacturer’s instructions. During the RNA purification step, 3.5 µL of synthetic cel-miR-39 spike-in control (Qiagen, Hilden, Germany) was added to each sample before RNA extraction as an exogenous control for normalization. The same volume of spike-in was added to all samples according to the provider’s recommendations and a previous publication [33]. Purified RNA was eluted in 14 μL of RNase-free water and immediately reverse transcribed, as described below.

miRNA expression was by two-step RT-qPCR using RT-primer specific assay in combination with TaqMan probes: hsa-miR-143-5p (Assay ID: 002146) and hsa-miR-223-3p (Assay ID: 002295) (Applied Biosystems; Foster City, CA, USA). Reverse transcription was performed using the TaqMan MicroRNA Reverse Transcription Kit (Applied Biosystems, Foster City, CA, USA) in a final reaction volume of 10 μL, including 1.5 μL of purified RNA. The RT reaction consisted of 30 min at 16 °C, 30 min at 42 °C, and 5 min at 85 °C.

One microliter of the RT reaction was used as the template for qPCR in a final reaction volume of 10 μL. PCR cycling conditions were initial denaturation at 95 °C for 10 min, followed by 45 cycles at 95 °C for 15 s, at 60 °C for 60 s, and at 72 °C for 1 s. PCR was performed using a 7900HT Fast Real-Time PCR System (Applied Biosystems, Foster City, CA, USA) with the Maxima Probe/ROX qPCR Master Mix (Thermo Scientific, Waltham, MA, USA). Relative miRNA expression levels were normalized with Ct values of the exogenous cel-miR-39 control, and values were calculated using the 2^−ΔΔCt^ formula. All Ct values for cel-miR-39 ranged from 20 to 22 cycles for total plasma miRNA isolations.

### 2.7. Statistical Analysis

Continuous variables were first assessed for normality with the Shapiro–Wilk test. According to their distribution, comparisons between groups were performed using either Student’s *t*-test or the Mann–Whitney U test. Categorical variables were analyzed using Pearson’s chi-square test.

Exploratory associations between miRNAs expression, added sugar intake, and cardiometabolic risk parameters were examined using Spearman’s correlation coefficients, as well as Student’s *t*-test.

Adjusted associations were investigated using multivariable linear regression models controlling for sex, age, BMI, and total energy intake. These models evaluated the associations of miRNA expression with cardiometabolic parameters and, separately, the associations of added sugar intake with both miRNA expression and cardiometabolic outcomes. For ease of interpretation, added sugar intake was entered as a continuous variable, with regression estimates expressed per 25 g/day increment. A two-sided *p* value < 0.05 was considered statistically significant. Statistical analyses were conducted using Stata version 19.5 (StataCorp LLC, College Station, TX, USA).

## 3. Results

### 3.1. Study Population Characteristics

Table 1 shows the children’s general characteristics and health outcome variables. The mean age was 9.197 ± 1.802 years. Males comprised 52% of the sample, with a higher proportion among children with overweight/obesity (~62%). BMI, BMI Z-score, waist and hip circumferences, insulin, HOMA-IR, total CHO, and TG levels were significantly higher in children with overweight/obesity compared with those with normal weight (*p* < 0.05), whereas HDL-C levels were lower in children with overweight/obesity (*p* < 0.001). No differences were observed in the participants’ age, glucose levels, LDL-C, and total energy or added sugar intake (*p* > 0.05).

### 3.2. Expression of miRNAs miR-143-5p and miR-223-3p in Children with High and Low Added Sugar Intake

**Figure 1:**
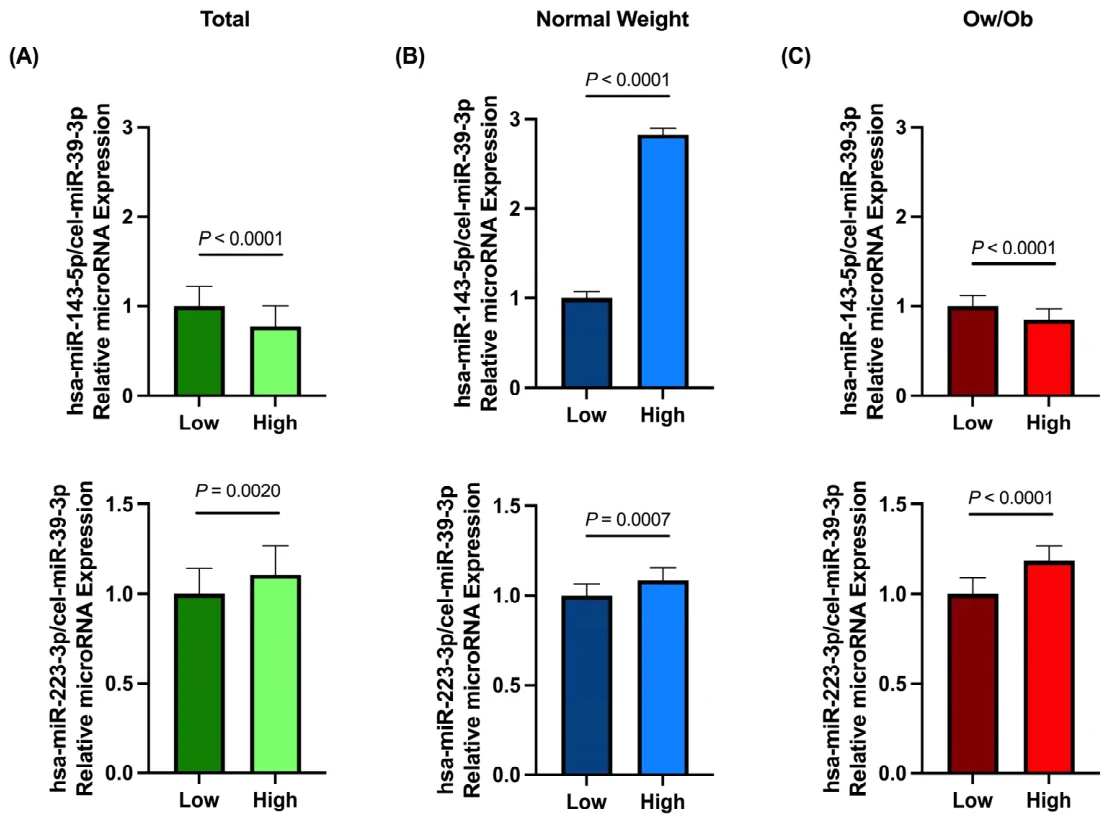
Plasma miRNA expression in children with NW and Ow/Ob stratified by low and high added sugar intake. (**A**) miR-143-5p and miR-223-3p expression in total children (green bars), (**B**) miR-143-5p and miR-223-3p expression in children with NW (blue bars), and (**C**) miR-143-5p and miR-223-3p expression in children with Ow/Ob (red bars). miRNA expression was measured by RT-qPCR using cel-miR-39 as the reference for the 2^−ΔΔCt^ method. Differences were tested using the unpaired *t*-test. Results are presented as mean ± SD.

High added sugar intake was associated with altered plasma miRNA expression in children. As shown in Figure 1A, miR-143-5p expression levels (1.000 ± 0.218 vs. 0.775 ± 0.2228; *p* < 0.001) were lower in children with high added sugar intake compared to those with low intake, whereas miR-223-3p expression levels (1.000 ± 0.142 vs. 1.106 ± 0.162; *p* = 0.002) were higher in children with high added sugar intake than in those with low intake.

Among children with NW, those with high added sugar intake showed higher expression levels of miR-143-5p (1.000 ± 0.072 vs. 2.823 ± 0.079; *p* < 0.001) (Figure 1B) and miR-223-3p (1.000 ± 0.064 vs. 1.084 ± 0.072; *p* < 0.001) (Figure 1B) than those with low added sugar intake. Among children with Ow/Ob, miR-143-5p expression levels (1.000 ± 0.112 vs. 0.845 ± 0.124; *p* < 0.001) were lower in children with high added sugar intake, whereas miR-223-3p expression levels (1.000 ± 0.088 vs. 1.184 ± 0.084; *p* < 0.001) were higher (Figure 1C).

**Figure 2:**
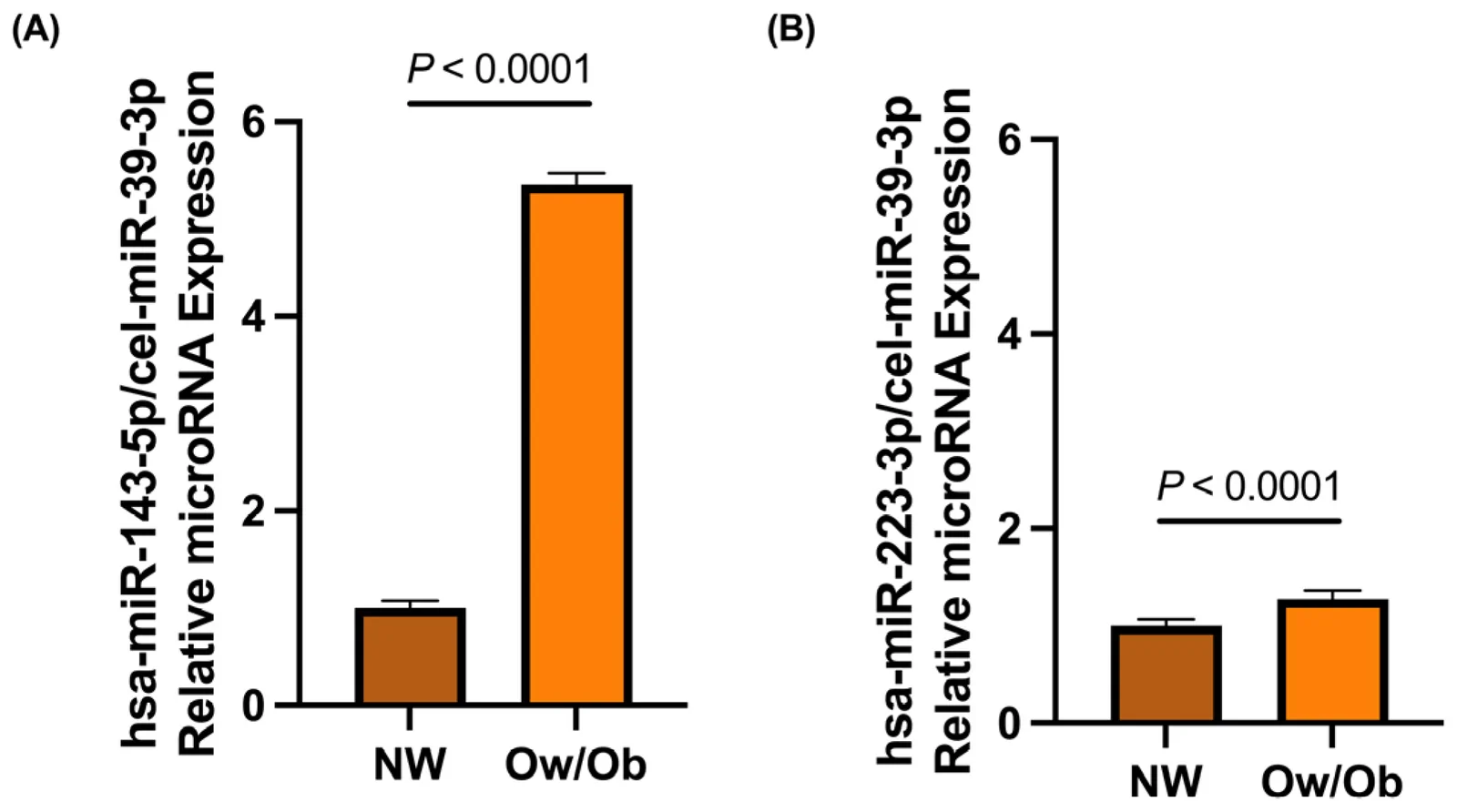
The plasma miRNA expression in children with NW and with Ow/Ob. (**A**) miR-143-5p expression and (**B**) miR-223-3p expression. miRNA expression was measured by RT-qPCR using cel-miR-39 as a reference for the 2^−ΔΔCt^ method. Differences were tested by an unpaired *t*-test. Results are presented as mean ± SD. NW: normal weight; Ow/Ob: Overweight/Obesity.

Subsequently, we evaluated miRNA expression levels in children according to nutritional status. Both miR-143-5p (1.000 ± 0.076 vs. 5.354 ± 0.118; *p* < 0.001) and miR-223-3p expression (1.000 ± 0.069 vs. 1.280 ± 0.086; *p* < 0.001) were higher in children with Ow/Ob compared with children with NW (Figure 2A,B).

**Figure 3:**
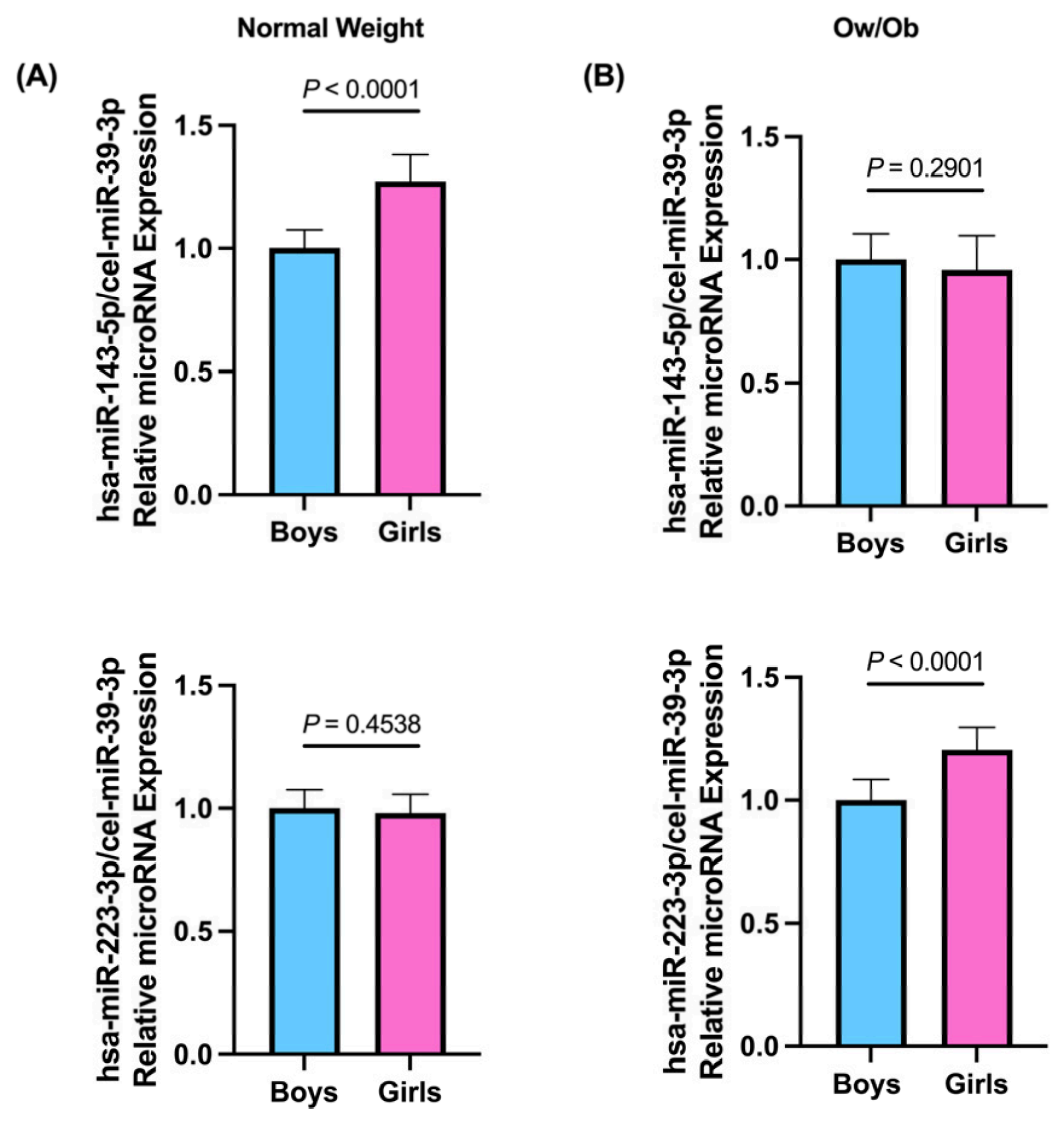
Plasma miRNA expression in boys and girls with NW and with Ow/Ob. (**A**) Children with normal weight. (**B**) Children with overweight/obesity (Ow/Ob). The upper panels show miR-143-5p expression, whereas the lower panels show miR-223-3p expression. miRNA expression was measured by RT-qPCR using cel-miR-39 as the reference for the 2^−ΔΔCt^ method. Differences were tested by an unpaired *t*-test. Results are presented as mean ± SD.

We further examined whether miRNA expression differed by sex and nutritional status. As shown in Figure 3A, among children with NW, miR-143-5p expression was higher in girls than in boys (1.000 ± 0.075 vs. 1.273 ± 0.107; *p* < 0.001), whereas miR-223-3p expression showed no significant differences (1.000 ± 0.075 vs. 0.981 ± 0.076; *p* = 0.453). Conversely, among children with Ow/Ob, miR-223-3p expression (1.000 ± 0.086 vs. 1.203 ± 0.091; *p* < 0.001) was higher in girls than in boys (Figure 3B), while miR-143-5p did not differ significantly among the groups (1.000 ± 0.106 vs. 0.958 ± 0.139; *p* = 0.290) (Figure 3B).

**Figure 4:**
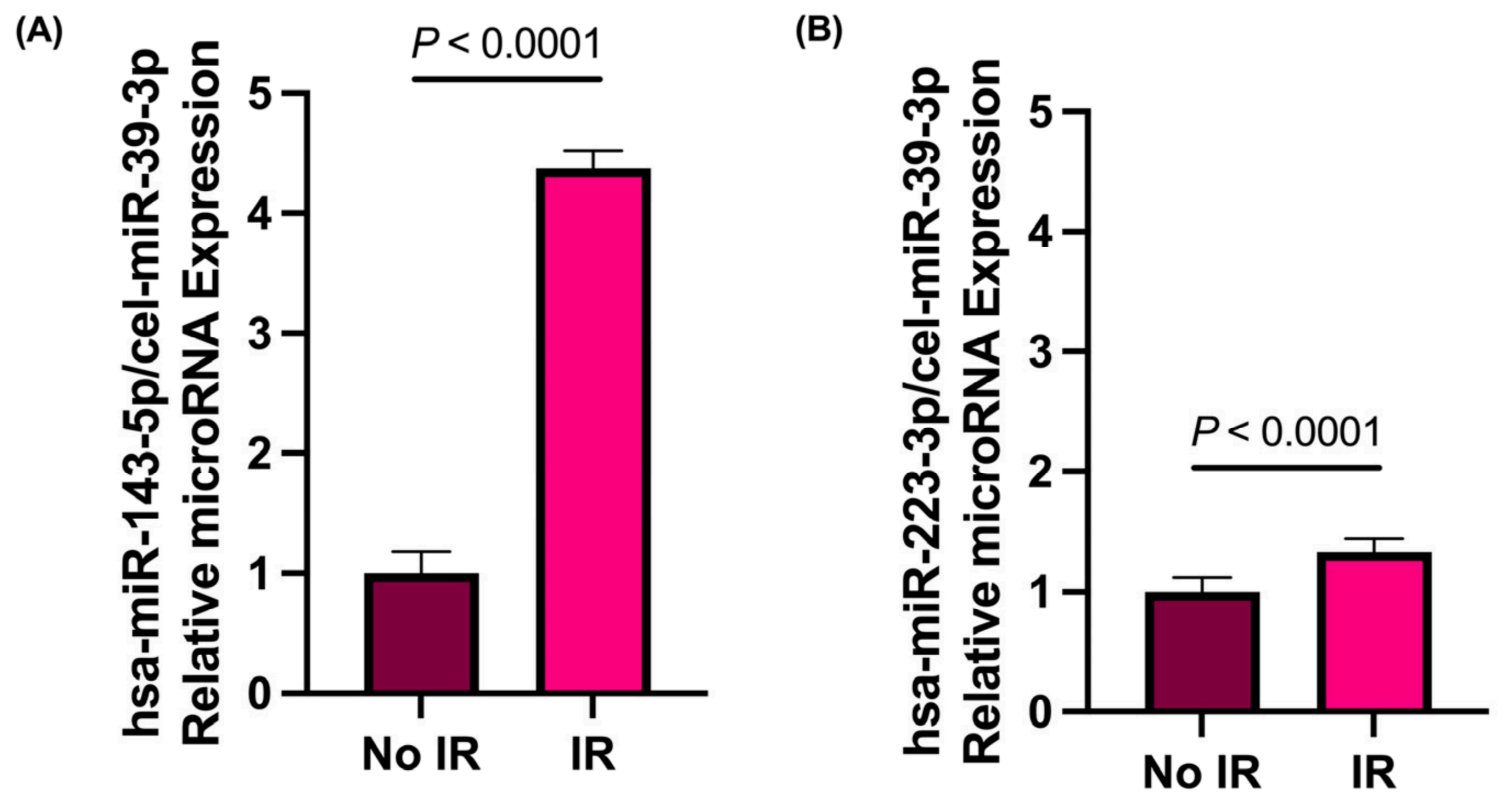
The plasma miRNA expression in children with and without insulin resistance. (**A**) miR-143-5p expression and (**B**) miR-223-3p expression. miRNA expression was measured by RT-qPCR using cel-miR-39 as a reference for the 2^−ΔΔCt^ method. Differences were tested by an unpaired *t*-test. Results are presented as mean ± SD. IR: Insulin resistance.

Finally, we evaluated plasma miRNA expression according to IR status. Based on the established cutoff, 52.75% of the children were classified as having IR. As shown in Figure 4A, children with IR showed higher miR-143-5p expression levels than those without IR (1.000 ± 0.180 vs. 4.371 ± 0.153; *p* < 0.001). Similarly, miR-223-3p expression levels were higher in children with IR (1.000 ± 0.118 vs. 1.329 ± 0.111; *p* < 0.001) (Figure 4B).

### 3.3. Associations Between Plasma miR-143-5p and miR-223-3p Expression and Cardiometabolic Risk Parameters

Exploratory Spearman’s correlations between plasma miR-143-5p and miR-223-3p expression and clinical and cardiometabolic risk parameters are presented in Supplementary Table S1. In these analyses, miR-143-5p expression was negatively correlated with BMI, BMI Z-score, waist circumference, and hip circumference (*p* < 0.05), and positively correlated with HDL-C levels (*p* < 0.05). Similarly, miR-223-3p expression was negatively correlated with hip circumference and positively correlated with added sugar intake (*p* < 0.05) (Supplementary Table S1).

However, as shown in Table 2, no significant associations were found between miR-143-5p or miR-223-3p expression and the evaluated cardiometabolic parameters (*p* > 0.05).

### 3.4. Associations Between Added Sugar Intake and miRNA Expression and Cardiometabolic Risk Parameters

Exploratory Spearman’s correlations between added sugar intake and cardiometabolic risk parameters are shown in Supplementary Table S2. According to these analyses, added sugar intake was positively correlated with miR-223-3p expression (*p* = 0.009) and glucose levels (*p* = 0.022). In the adjusted models, added sugar intake was positively associated with miR-223-3p expression (β = 0.188; 95% CI: 0.022–0.354; *p* = 0.027) and with glucose levels (β = 1.944; 95% CI: 0.133–3.755; *p* = 0.036) (Table 3). No significant associations were found for the remaining variables.

## 4. Discussion

The findings of this study suggest that added sugar intake is associated with alterations in circulating miRNA expression in children. Specifically, children with high added sugar intake exhibited lower plasma miR-143-5p expression and higher miR-223-3p expression compared with those with lower intakes. In addition, both miR-143-5p and miR-223-3p expression levels were higher in children with overweight/obesity and in those with insulin resistance. However, after adjustment, only added sugar intake remained positively associated with miR-223-3p expression and fasting glucose concentrations, whereas neither miRNA was associated with cardiometabolic risk parameters. Together, these findings suggest that added sugar intake may be associated with specific circulating miRNAs involved in metabolic regulation. However, given the cross-sectional design of this study, these associations should be interpreted cautiously and confirmed in larger longitudinal studies.

Childhood obesity is an emerging global health concern, as overweight and obesity during childhood have been shown to persist into adulthood [1], and higher added sugar intake has been associated with weight gain and an increased risk of obesity in children [6,7]. miRNAs are important regulators of metabolic balance [34]. Among these miRNAs, miR-143-5p and miR-223-3p have been implicated in metabolic regulation. miR-143 is conserved between humans and rats, and several studies have shown its important role in lipid metabolism, adipogenesis, and IR [19]. miR-223-3p has also been implicated in metabolic regulation and IR [35]. In our study, children with normal weight and high added sugar intake showed higher plasma miR-143-5p expression, whereas lower expression was observed in children with overweight/obesity. In the case of miR-223-3p, children with normal weight and overweight/obesity and high added sugar intake showed higher plasma miR-223-3p expression. A previous study showed that high-fructose intake increased miR-143-5p expression and reduced miR-223-3p expression in extracellular vesicles from plasma in murine models [25]. Conversely, high sucrose intake did not alter plasma levels [24].

After adjusting for sex, age, BMI, and total energy intake, added sugar intake in our study was positively associated with plasma miR-223-3p expression levels in children. Specifically, each 25 g/day increase in added sugar intake was associated with a 0.188-unit increase in plasma miR-223-3p expression, corresponding to an approximately 18.8% relative increase, although the biological significance of this association remains to be determined. In addition, each 25 g/day increase in added sugar intake was associated with an average 1.944 mg/dL increase in fasting glucose concentrations, supporting the relationship between higher added sugar consumption and early metabolic alterations in children. Taken together, these findings suggest a potential relationship between chronic added sugar intake and circulating miRNA expression. These findings suggest that miR-223-3p expression may be associated with dietary sugar intake.

Additionally, plasma miR-143-5p expression was upregulated in children with overweight/obesity. This miRNA was negatively correlated with BMI, BMI Z-score, waist circumference, hip circumference and positively correlated with HDL-C levels. Although these findings may appear discordant, they reflect different analytical approaches, as the group comparison evaluated predefined nutritional status categories, whereas the correlation analysis assessed anthropometric variables continuously across the entire study population. This pattern may also reflect heterogeneity in miR-143-5p expression within the overweight/obesity group. Similar results were found for serum miR-143-5p in children, adolescents, and adults with obesity [36,37]. In experimental models of high-fat diet-induced obesity, miR-143-5p expression was associated with body weight gain, mesenteric adipose tissue gain, and key adipogenesis markers [38]. Moreover, miR-143-5p promotes adipogenesis through the MAP2K5-ERK5 regulatory pathway [39]. Its inhibition decreases the expression of adipocyte-specific genes, such as PPAR-γ, aP2, and GLUT-4, as well as triglyceride levels. However, these associations were not maintained after adjustment.

miR-223-3p plasma expression was upregulated in children with overweight/obesity and negatively correlated with hip circumference. A study reported that plasma miR-223-3p expression was upregulated in children and adolescents with obesity and was inversely associated with obesity [40], suggesting that higher levels of this miRNA may be related to a lower probability of obesity. However, opposite results were observed in adults with obesity [37] and in patients with T2D [41]. In addition, miR-223-3p expression was upregulated in human omental adipose tissue from patients with obesity [42]. Functionally, miR-223-3p attenuates the pro-inflammatory activation of macrophages [43] and regulates NLRP3 and IL-1β production in mouse adipose tissue [44]. In vitro studies showed that TNF-α-stimulated preadipocytes, mimicking the inflammatory environment present in obesity, exhibited reduced secretion of this miRNA and increased intracellular accumulation, impairing glucose and lipid metabolism [41]. Moreover, studies have reported that miR-223-3p enhances adipogenic differentiation of mesenchymal stem cells [45] and human adipocytes [46]. Therefore, the altered expression of miR-143-5p and miR-223-3p observed in children with overweight/obesity may be consistent with adipose tissue dysfunction, although this interpretation requires further investigation. However, neither miRNA was associated with cardiometabolic risk parameters in adjusted analyses.

No overall differences in plasma miR-143-5p and miR-223-3p expression were observed between boys and girls. However, when children were stratified by nutritional status, plasma miR-143-5p expression was upregulated in girls with normal weight, consistent with previous findings reported in boys and girls with normal weight [36]. Although the underlying mechanisms are not fully understood, this finding may be related to sex-specific differences in hormonal regulation, body composition, and pubertal development, which could modulate circulating miRNA profiles. Moreover, plasma miR-223-3p expression was upregulated in girls with overweight/obesity. Although sex-specific evidence for miR-223-3p in children is limited, altered circulating miR-223-3p levels have been reported in pediatric obesity [40], and sex-related differences in miRNA profiles have been described in adolescents with obesity [47]. Therefore, these findings suggest that the influence of sex on circulating miRNA expression may depend on nutritional and metabolic status. Nevertheless, these results should be interpreted cautiously, as sex-related differences in circulating miRNAs appear to be miRNA-specific and may vary according to population characteristics, age, metabolic status, and the biological matrix analyzed [47,48].

On the other hand, our study showed higher plasma miR-143-5p expression in children with IR. Previous in vitro studies demonstrated that FFAs, leptin, and resistin, key components of the obesogenic inflammatory microenvironment involved in IR, inhibited miR-143 expression in human adipocytes [49]. In addition, bone marrow macrophage-derived exosomal miR-143-5p has been shown to induce IR in hepatocytes by repressing MKP5 [50]. In our study, the expression levels of miR-223-3p were increased in children with IR. Similar findings have been reported in serum from adolescents with obesity and IR [51]. In contrast, a study conducted in adults with IR found that plasma miR-223-3p expression levels were lower and negatively correlated with the adipose tissue IR index [41]. In vitro evidence indicates that increased miR-223-3p expression reduces GLUT4 expression in human adipocytes [52]. Moreover, miR-223-3p reduced the expression levels of NLRP3 mRNA, Bax and NLRP3 protein, thereby inhibiting apoptosis in endothelial cells [23]. Although both miR-143-5p and miR-223-3p were expressed at higher levels in children with IR, neither miRNA was associated with insulin, HOMA-IR, or glucose concentrations after adjustment for age, sex, BMI, and total energy intake. Therefore, these findings should be interpreted cautiously and may indicate that the roles of miR-143-5p and miR-223-3p in obesity-related IR are complex and may vary across tissues, cell types, and biological compartments analyzed.

The present study has some limitations. First, its cross-sectional design precludes establishing causal relationships between added sugar intake, circulating miRNA expression, and cardiometabolic outcomes. Second, the relatively modest sample size may have limited the ability to detect weaker associations. Third, although age was included as a covariate in the adjusted regression analyses, pubertal stage was not assessed. Because the study included children aged 6–12 years, pubertal maturation may have influenced body composition, insulin sensitivity, glucose metabolism, and circulating miRNA expression. Therefore, these findings should be interpreted as observational associations and confirmed in larger prospective studies incorporating pubertal assessment.

## 5. Conclusions

High added sugar intake was associated with altered plasma miRNA expression in children, characterized by lower miR-143-5p expression and higher miR-223-3p expression. In addition, both miR-143-5p and miR-223-3p expression levels were higher in children with overweight/obesity and in those with insulin resistance. After adjustment, added sugar intake was positively associated with miR-223-3p expression and fasting glucose concentrations, whereas neither miRNA was associated with cardiometabolic risk parameters. These findings suggest that circulating miRNAs may be associated with dietary added sugar intake; however, larger longitudinal studies are needed to clarify their biological significance and potential role in metabolic health.

## Figures and Tables

**Table 1:** Demographic, clinical, and cardiometabolic parameters.

	Total (*n* = 91)	NW (*n* = 44)	Ow/Ob (*n* = 47)	*p*-Value
Age (years)	9.197 ± 1.802	8.931 ± 1.822	9.446 ± 1.766	0.174 ^a^
Sex, *n* (%)				
Male vs. Female	47 (51.650)	18 (40.910)	29 (61.700)	0.047 ^b^
BMI (kg/m^2^)	18.653 (16.005, 25.716)	16.105 (15.422, 17.400)	25.414 (23.804, 28.268)	<0.001 ^c^
BMI Z-score	1.160 (0.140, 2.160)	0.125 (−0.270, 0.475)	2.160 (1.930, 2.370)	<0.001 ^c^
Waist (cm)	70 (59, 85)	60 (55, 63)	85 (79, 92)	<0.001 ^c^
Hip (cm)	81 (70, 93)	70 (65, 77)	93 (84, 100)	<0.001 ^c^
CHO (mg/dL)	156.875 ± 27.853	150.047 ± 20.807	163.268 ± 32.044	0.022 ^a^
HDL-C (mg/dL)	45.240 ± 9.723	51.325 ± 8.669	39.544 ± 6.794	<0.001 ^a^
LDL-C (mg/dL)	80.657 ± 27.454	75.061 ± 22.872	85.895 ± 30.458	0.059 ^a^
TG (mg/dL)	145.500 (84.600, 221.700)	91.500 (69.800, 145.400)	196.900 (144.800, 240.500)	<0.001 ^c^
Glucose (mg/dL)	87.973 ± 8.703	87.629 ± 7.811	88.295 ± 9.536	0.717 ^a^
Insulin (μU/mL)	13.300 (8.010, 24.800)	8.69 (6.450, 11.615)	23.890 (15.770, 32.920)	<0.001 ^c^
HOMA-IR	3.066 (1.770, 5.253)	1.820 (1.407, 2.398)	5.006 (3.461, 6.872)	<0.001 ^c^
≥3.16, *n* (%)	48 (52.750)	9 (20.450)	39 (82.980)	<0.001 ^b^
Total energy intake (Kcal)	2542.183 ± 799.027	2641.665 ± 857.360	2449.051 ± 737.342	0.252 ^a^
Added sugar intake (g)	65.103 (39.137, 90.733)	67.566 (45.432, 88.746)	63.282 (37.540, 86.427)	0.356 ^c^
≥50 g, *n* (%)	54 (59.340)	29 (65.910)	25 (53.190)	0.217 ^b^

BMI: Body mass index; CHO: Cholesterol; HDL-C: High-density lipoprotein cholesterol; LDL-C: Low-density lipoprotein cholesterol; TG: Triglycerides; HOMA-IR: Homeostatic model assessment of IR. Results are expressed as mean ± standard deviation for parametric data or as median (interquartile range) for non-parametric data. a Student’s *t* test; b Chi-square test; c Mann–Whitney test. *p* values < 0.05 were considered statistically significant.

**Table 2:** Association of miRNA expression and cardiometabolic risk parameters.

	miR-143-5p			miR-223-3p		
	β	95% CI	*p*-Value	β	95% CI	*p*-Value
CHO (mg/dL)	0.002	−0.004, 0.008	0.470	0.000	−0.006, 0.006	0.923
HDL-C (mg/dL)	0.009	−0.015, 0.034	0.439	−0.000	−0.025, 0.023	0.958
LDL-C (mg/dL)	−0.001	−0.007, 0.005	0.709	−0.001	−0.008, 0.004	0.564
TG (mg/dL)	0.002	−0.000, 0.004	0.089	0.001	−0.000, 0.004	0.194
Glucose (mg/dL)	0.010	−0.009, 0.029	0.302	0.002	−0.017, 0.022	0.792
Insulin (μU/mL)	0.007	−0.005, 0.020	0.271	0.010	−0.003, 0.023	0.146
HOMA-IR	0.035	−0.022, 0.093	0.226	0.044	−0.015, 0.104	0.141

CHO: Cholesterol; HDL-C: High-density lipoprotein cholesterol; LDL-C: Low-density lipoprotein cholesterol; TG: Triglycerides; HOMA-IR: Homeostatic model assessment of insulin resistance. β values represent unstandardized coefficients derived from linear regression models with miRNA expression as dependent variables, adjusted for sex, age, BMI and total energy intake. *p* values < 0.05 were considered statistically significant.

**Table 3:** Association of the added sugar intake with miRNA expression and cardiometabolic risk parameters.

	Added Sugar (g)		
	β	95% CI	*p*-Value
miR-143-5p	0.109	−0.056, 0.275	0.194
miR-223-3p	0.188	0.022, 0.354	0.027
CHO (mg/dL)	−3.125	−8.783, 2.533	0.275
HDL-C (mg/dL)	0.516	−0.991, 2.025	0.497
LDL-C (mg/dL)	−4.235	−9.796, 1.325	0.134
TG (mg/dL)	2.966	−12.038, 17.971	0.695
Glucose (mg/dL)	1.944	0.133, 3.755	0.036
Insulin (μU/mL)	1.481	−1.172, 4.135	0.270
HOMA-IR	0.443	−0.158, 1.045	0.146

CHO: Cholesterol; HDL-C: High-density lipoprotein cholesterol; LDL-C: Low-density lipoprotein cholesterol; TG: Triglycerides; HOMA-IR: Homeostatic model assessment of IR. β values represent unstandardized coefficients derived from linear regression models with added sugar intake as the independent variable, adjusted for sex, age, BMI, and total energy intake. Results are presented as β and 95% CI. *p* values < 0.05 were considered statistically significant.

## Data Availability

The raw data supporting the conclusions of this article will be made available by the authors on request due to ethical restrictions and the need to protect participant confidentiality in accordance with institutional guidelines.

## Supplementary Materials

The following supporting information can be downloaded at https://www.mdpi.com/article/10.3390/biom16081100/s1, Table S1: Spearman’s correlation coefficients between miR-143-5p, miR-223-3p clinical and cardiometabolic parameters; Table S2: Spearman’s correlation coefficients between added sugar and cardiometabolic parameters.

## Abbreviations

The following abbreviations are used in this manuscript:

3′UTR	3′ untranslated region
BMI	Body mass index
BMI Z-score	Body mass index Z-score
cDNA	Complementary DNA
CHO	Cholesterol
ENSANUT	National Health and Nutrition Survey
FFAs	Free fatty acids
FFQ	Food frequency questionnaire
GLUT-4	Glucose transporter type 4
HDL-C	High-density lipoprotein cholesterol
HOMA-IR	Homeostatic model assessment of insulin resistance
IL-1β	Interleukin-1 beta
IR	Insulin resistance
LDL-C	Low-density lipoprotein cholesterol
MAP2K5-ERK5	Mitogen-activated protein kinase kinase 5–extracellular signal-regulated kinase 5
miR-143-5p	microRNA-143-5p
miR-223-3p	microRNA-223-3p
miRNA	microRNA
mRNA	Messenger RNA
NLRP3	NOD-like receptor family pyrin domain containing 3
NW	Normal weight
Ow/Ob	Overweight/obesity
PPAR-γ	Peroxisome proliferator-activated receptor gamma
RNA	Ribonucleic acid
T2D	Type 2 diabetes
TG	Triglycerides
TNF-α	Tumor necrosis factor alpha
WHO	World Health Organization
