# Supplementary material for: Added Sugar Intake Is Associated with Altered Plasma miR-143-5p and miR-223-3p Expression and Fasting Glucose Concentrations in Children from Mexico City
Source: Biomolecules. 2026 Jul 28;16(8):1100. doi: 10.3390/biom16081100 (PMC13511039; doi:10.3390/biom16081100)
Supplement: Supplementary file 1 [file biomolecules-16-01100-s001.zip › biomolecules-4457091-supplementary.pdf]

Table S1. Spearman's correlation coefficients between miR-143-5p, miR-223-3p clinical and cardiometabolic parameters.

	miR-143-5p (n= 91)		miR-223-3p (n= 91)	
	**Rho**	**P-value**	**Rho**	**P-value**
BMI (kg/m2)	-0.2601	0.0158	-0.1177	0.2660
BMI Z-score	-0.2249	0.0375	-0.0306	0.7733
Waist (cm)	-0.2236	0.0387	-0.1534	0.1463
Hip (cm)	-0.2757	0.0104	-0.2175	0.0385
CHO (mg/dL)	0.0550	0.6140	-0.0001	0.9990
HDL-C (mg/dL)	0.2719	0.0115	0.1202	0.2560
LDL-C (mg/dL)	-0.0522	0.6326	-0.0620	0.5588
TG (mg/dL)	0.0662	0.5438	0.1126	0.2873
Glucose (mg/dL)	0.1454	0.1813	0.0474	0.6547
Insulin (µU/mL)	-0.1119	0.3044	0.0469	0.6585
HOMA-IR	-0.1139	0.2957	0.0308	0.7712
Total energy (Kcal)	0.0743	0.4957	0.0486	0.6469
Added sugar (g)	0.1351	0.2146	0.2329	0.0265

BMI: Body mass index; CHO: Cholesterol; HDL-C: High-density lipoprotein cholesterol; LDL-C: Low-density lipoprotein cholesterol; TG: Triglycerides; HOMA-IR: Homeostatic model assessment of insulin resistance. Rho values correspond to the Spearman correlation coefficients. P values < 0.05 were considered statistically significant.

Table S2. Spearman's correlation coefficients between added sugar and cardiometabolic parameters.

	Added sugar (g) (n= 91)	
	**Rho**	**P-Value**
miR-143-5p	0.1351	0.2146
miR-223-3p	0.2329	0.0265
BMI (kg/m^2)	-0.0970	0.3736
BMI Z-score	-0.1346	0.2163
Waist (cm)	-0.0254	0.8163
Hip (cm)	-0.0871	0.4243
CHO (mg/dL)	-0.0707	0.5173
HDL-C (mg/dL)	0.0157	0.8856
LDL-C (mg/dL)	-0.0451	0.6793
TG (mg/dL)	-0.0288	0.7920
Glucose (mg/dL)	0.2470	0.0221
Insulin (µU/mL)	-0.0135	0.9019
HOMA-IR	0.0277	0.8001

BMI: Body mass index; CHO: Cholesterol; HDL-C: High-density lipoprotein cholesterol; LDL-C: Low-density lipoprotein cholesterol; TG: Triglycerides; HOMA-IR: Homeostatic model assessment of insulin resistance. Rho values correspond to the Spearman correlation coefficients. P values < 0.05 were considered statistically significant.
